# A Recombinant Peptide Device Combined with Adipose Tissue-Derived Stem Cells Enhances Subcutaneous Islet Engraftment

**DOI:** 10.3390/cells13060499

**Published:** 2024-03-13

**Authors:** Takahiro Mizui, Akiko Inagaki, Yasuhiro Nakamura, Takehiro Imura, Satomi Suzuki Uematsu, Shigehito Miyagi, Takashi Kamei, Michiaki Unno, Kimiko Watanabe, Masafumi Goto

**Affiliations:** 1 Department of Surgery, Tohoku University Graduate School of Medicine, Sendai 980-0872, Japan; tmizui@ncc.go.jp (T.M.); satomi@med.tohoku.ac.jp (S.S.U.); msmsmiyagi@yahoo.co.jp (S.M.); tkamei@surg.med.tohoku.ac.jp (T.K.); m_unno@surg.med.tohoku.ac.jp (M.U.); 2 Department of Hepatobiliary and Pancreatic Surgery, National Cancer Center Hospital, Tokyo 104-0045, Japan; 3 Division of Transplantation and Regenerative Medicine, Tohoku University Graduate School of Medicine, Sendai 980-8575, Japan; akiko.inagaki.b1@tohoku.ac.jp (A.I.); takehiro.imura.b1@tohoku.ac.jp (T.I.); kimiko.watanabe.d8@tohoku.ac.jp (K.W.); 4 Division of Pathology, Graduate School of Medicine, Tohoku Medical and Pharmaceutical University, Sendai 983-8536, Japan; yasu-naka@tohoku-mpu.ac.jp

**Keywords:** subcutaneous space, islets, transplantation, recombinant peptide (RCP) device, adipose tissue-derived stem cells (ADSCs)

## Abstract

Subcutaneous space has been considered an attractive site for islet graft transplantation; however, the oxygen tension and vascularization are insufficient for islet graft survival. We investigated whether subcutaneous pre-implantation of a recombinant peptide (RCP) device with adipose tissue-derived stem cells (ADSCs) enhanced subcutaneous islet engraftment. RCP devices with/without syngeneic ADSCs were pre-implanted into the subcutaneous space of C57BL/6 mice. Syngeneic islets (300 or 120 islet equivalents (IEQs)) were transplanted into the pre-treated space after diabetes induction using streptozotocin. The cure rates of groups in which RCP devices were implanted four weeks before transplantation were significantly better than the intraportal transplantation group when 300 IEQs of islets were transplanted (*p* < 0.01). The blood glucose changes in the RCP+ADSCs-4w group was significantly ameliorated in comparison to the RCP-4w group when 120 IEQs of islets were transplanted (*p* < 0.01). Immunohistochemical analyses showed the collagen III expression in the islet capsule of the RCP+ADSCs-4w group was significantly enhanced in comparison to the RCP-4w and RCP+ADSCs-d10 groups (*p* < 0.01, *p* < 0.01). In addition, the number of von Willebrand factor-positive vessels within islets in the RCP+ADSCs-4w group was significantly higher than the RCP-4w group. These results suggest that using ADSCs in combination with an RCP device could enhance the restoration of the extracellular matrices, induce more efficient prevascularization within islets, and improve the graft function.

## 1. Introduction

Pancreatic islet transplantation, in which isolated islet grafts are injected into the liver through the portal vein, has become an effective clinical treatment option for complicated type 1 diabetes [1]. However, there are still several issues to be resolved regarding this procedure, including damage to the islet grafts, due to strong innate immune reactions [2]; the risks of bleeding, embolism [3], and portal hypertension [4]; and the difficulty associated with monitoring and removing islet grafts [5]. Thus, alternative extrahepatic transplant sites should be investigated to overcome these limitations.

The subcutaneous space has many advantages as a transplant site for islet grafts. It is easily accessible for the implantation and removal of islet grafts and can be routinely monitored by biopsy with minimal invasiveness [6]. However, islet transplantation into the unmodified subcutaneous space results in the need for huge amounts of islet grafts to control diabetes, because the subcutaneous space is too poorly vascularized to provide sufficient oxygen and nutrients for the survival and function of the islet grafts [7,8]. In this regard, the optimization of prevascularization procedures at the subcutaneous transplant site before transplantation is essential for improving subcutaneous islet engraftment.

Recently, stem cells have attracted considerable attention for their ability to support cell survival. Among the different types of stem cells, adipose tissue-derived stem cells (ADSCs) can be obtained in large quantities using a relatively simple, non-invasive, and inexpensive procedure [9]. ADSCs have been reported to improve the revascularization process through the secretion of antiapoptotic and angiogenic cytokines and by direct differentiation into endothelial cells [10,11]. Thus, ADSCs can potentially allow the improvement of subcutaneous islet engraftment with enhanced prevascularization.

We previously reported the efficacy of the engraftment of islets subcutaneously transplanted using a recombinant peptide (RCP: alpha-1 sequence of recombinant collagen type I supplemented with a 12 RGD [Arg-Gly-Asp] motifs in 1 molecule) device as a novel subcutaneous scaffold [12]. In this report, we demonstrated that four-week placement of the RCP device showed comparable outcomes to standard intraportal islet transplantation. However, immunohistochemical analyses showed that there was no significant difference in the number of von Willebrand factor (vWF)-positive vessels between the RCP group and other groups. Based on these results, in the present study, we investigated whether the addition of ADSCs in combination with an RCP device could induce more efficient prevascularization and improve subcutaneous islet engraftment using a marginal dose of islets in a syngeneic mouse model.

## 2. Materials and Methods

### 2.1. Animals

All of the animals used in the present study were handled in accordance with the Guide for the Care and Use of Laboratory Animals published by the National Institutes of Health [13] and the guidelines for animal experiments and related activities at Tohoku University (protocol ID: 2018 MdA-175). C57BL/6 mice (male, 8 to 12 weeks of age) (Japan SLC, Inc., Shizuoka, Japan) were used as both donors and recipients.

Luciferase transgenic (Luc-Tg) Lewis rats (female, 11 weeks of age) were provided by Prof Eiji Kobayashi (The Jikei University school of Medicine, Tokyo, Japan) and were bred at Tohoku University for in vivo bioluminescent imaging. These Luc-Tg rats were used as donors, and Balb/c nude mice (male, 12 weeks of age) (Japan SLC) were used as recipients.

### 2.2. The Isolation and Culture of ADSCs

ADSCs were isolated from the adipose tissues of male C57BL/6 mice (10–12 weeks of age) (Japan SLC) or female Luc-Tg rats as reported previously [14,15]. The adipose tissues were obtained from the subcutaneous fat around the lower abdomen, washed with phosphate-buffered saline (PBS) containing 100 units/mL penicillin and 100 μg/mL streptomycin (Thermo Fisher Scientific Inc., Waltham, MA, USA), and cut into small pieces. The adipose tissues were then digested in Hank’s balanced salt solution (HBSS) containing 1 mg/mL collagenase type II (Sigma-Aldrich, St. Louis, MO, USA) and 100 units/mL penicillin and 100 μg/mL streptomycin at 37 °C for 30 min using a shaker at 120 rpm. After digestion, the tissue suspension was filtered through a 70 µm cell strainer (Corning Inc., New York, NY, USA) to remove tissue clumps and centrifuged at 400× *g* for 5 min at room temperature. The pellet was washed twice in HBSS containing 10% fetal bovine serum (FBS; Equitech-Bio Inc., Kerrville, TX, USA) and suspended in ADSC growth medium (ADSC-1; Kojin-Bio Co., Ltd., Sakado, Japan) containing 100 units/mL penicillin and 100 μg/mL streptomycin. Finally, the ADSCs were seeded onto cell culture flasks (2.0 × 10^5^ cells/cm^2^) and incubated at 37 °C with 5% CO_2_. ADSCs at passage 1 were used for experiments.

### 2.3. Flow Cytometry

Flow cytometry was performed to characterize the phenotypes of ADSCs. The isolated ADSCs (1.0 × 10^5^ cells) of C56BL/6 mice were labeled with fluorescence isothiocyanate-conjugated monoclonal antibodies against CD31, CD90, CD44, CD45, and Ly6A/E (Sca-1) (BD Biosciences, Franklin Lakes, NJ, USA) for 60 min on ice. Then, the labeled cells were analyzed for cell surface antigen expression using a BD Accuri^TM^ C6 Flow Cytometer (BD Biosciences). The raw data were further analyzed using the BD Accuri^TM^ C6 Software (BD Biosciences).

### 2.4. In Vivo Bioluminescence Imaging of ADSCs Seeded on RCP Devices

The ADSCs isolated and cultured from the inguinal adipose tissue of Luc-Tg rats were seeded on RCP devices (kindly provided by Fujifilm Corporation [cellnest; Tokyo, Japan]). Devices with ADSCs were implanted in the subcutaneous space of Balb/c nude mice. To detect the luciferase expression of the transplanted ADSCs, 150 mg/kg of D-luciferin potassium salt (Promega Corp., Madison, WI, USA) dissolved in PBS was intraperitoneally infused. Luciferase imaging was obtained using the IVIS Spectrum in vivo imaging system (PerkinElmer Inc., Waltham, MA, USA) and analyzed using the Living Image Software imaging system (PerkinElmer Inc.). To confirm the presence of viable ADSCs on the RCP device, we examined the luciferase expression 0, 10, 21, and 28 days after implantation.

### 2.5. Preparation and Implantation of RCP Devices with/without ADSCs

To efficiently induce neovascularization at the subcutaneous transplant site, as previously described [12], we subcutaneously implanted the RCP device before transplantation. The RCP device (diameter, 16 mm; thickness, 1 mm) was kindly provided by Fujifilm Corporation (cellnest™; Tokyo, Japan). It was prepared using a previously reported method [16,17]. The RCP has been explored based on the alpha-1 sequence of human collagen type I; 12 RGD (Arg-Gly-Asp) motifs were contained in 1 molecule. 

The RCP devices were incubated in ADSC-1 medium for two days to remove air bubbles from the devices. The ADSCs were trypsinized with 0.05% trypsin and 0.53 mM ethylenediaminetetraacetic acid (Thermo Fisher Scientific Inc.) for 3 min at 37 °C, counted, resuspended in ADSC-1, and then 1.0 × 10^5^ cells of ADSCs were seeded on both front and back sides of the devices according to the previous report [15] and our preliminary trials. The devices seeded with ADSCs were cultured overnight at 37 °C under 5%CO_2_. The viability of the ADSCs on the devices was evaluated by fluorescein diacetate (FDA; Sigma-Aldrich) and propidium iodide (PI; Merck Millipore, Darmstadt, Germany) staining. For FDA/PI staining, the devices were submerged in 10 µg/mL FDA and 5 µg/mL PI for 5 min and then observed under an inverted phase contrast fluorescence microscope (BZ-9000; KEYENSE, Tokyo, Japan). On the next day, RCP devices with/without ADSCs were implanted into the left dorsal subcutaneous space of the recipient mice. The recipient mice were divided into the following four groups: RCP+ADSC-4w, RCP-4w, RCP+ADSC-d10, and RCP-d10 groups. In the RCP+ADSC-4w and RCP-4w groups, RCP devices were implanted with/without ADSCs four weeks before transplantation, whereas in the RCP+ADSC-d10 and RCP-d10 groups, the devices were implanted 10 days before transplantation.

### 2.6. Islet Isolation and Transplantation

Islet isolation and culturing were performed as previously described [18]. The islets were cultured in Roswell Park Memorial Institute-1640 medium containing 5.5 mmol/L glucose and 10% FBS at 37 °C in 5% CO_2_ and humidified air overnight before transplantation.

After the removal of the RCP devices with/without ADSCs, 300 or 120 islet equivalents (IEQs) of syngeneic mouse islets were transplanted into the pre-treated space using a gastight syringe (Hamilton Co., Reno, NV, USA). On the other hand, in the intraportal transplantation (Ipo-Tx) group, 300 IEQs of syngeneic mouse islets in a total volume of 300 µL were infused into the recipient liver through the portal vein using a 27-gauge Surshield (TERUMO, Inc., Tokyo, Japan).

### 2.7. The Induction and Diagnosis of Diabetes in the Recipients

Diabetes was induced by the intravenous injection of 170 mg/kg streptozotocin (Sigma-Aldrich) seven days before subcutaneous or intraportal islet transplantation. Mice whose non-fasting blood glucose levels were ≥400 mg/dL on two consecutive measurements were considered diabetic. Serial blood glucose levels were determined, and recipients whose non-fasting blood glucose levels were <200 mg/dL on two consecutive measurements were considered to be cured.

### 2.8. Intraperitoneal Glucose Tolerance Test

An intraperitoneal glucose tolerance test (IPGTT) was performed 29 to 31 days after islet transplantation. D-glucose (1.0 g/kg) was intraperitoneally infused, and blood glucose concentrations were determined before and 5, 10, 15, 20, 25, 30, 45, 60, 90, and 120 min after the injection of glucose. The results of the IPGTT were evaluated based on the area under the curve (AUC).

### 2.9. Immunohistochemical Staining

The recipient tissues at the subcutaneous transplant site were harvested, fixed with 4% paraformaldehyde, and embedded in paraffin for immunohistochemical staining seven days after transplantation. Immunohistochemical staining was performed using an In Situ Apoptosis Detection Kit (Trevigen, Inc., Gaithersburg, MD, USA) for TdT-mediated dUTP nick end labeling (TUNEL) staining, anti-vWF antibody (AB7356; MerckMillipore, Darmstadt, Germany), anti-insulin antibody (IS002; Dako, Glostrup, Denmark), anti-laminin antibody (ab11575; Abcam, Cambridge, UK), anti-fibronectin antibody (ab2413; Abcam), anti-collagen III antibody (ab7778; Abcam), anti-collagen IV antibody (ab6586; Abcam), and an EnVision System-HRP (Dako). For the evaluation of neovascularization and apoptosis, the TUNEL- and vWF-positive cells in the islet and interstitial areas were counted [19]. In fibronectin staining, “positive” was defined as marked immunopositivity detectable in the interstitial area, whereas in laminin, collagen III, and collagen IV staining, it was defined as distinct immunopositivity detectable in the fibrous capsule around the islets [20]. More than 58 sections from each experimental group (*n* = 6 or 7) were evaluated by a pathologist using a blind method.

### 2.10. Statistical Analyses

All data are expressed as the mean ± standard deviation. The statistical analyses were performed using the JMP pro 14 (SAS Institute Inc., Cary, NC, USA). The changes in the blood glucose levels were analyzed by two-way analysis of variance, and a Tukey–Kramer test was used for post hoc comparisons between the groups. The AUC of the IPGTT was analyzed by the Mann–Whitney test and the number of vWF-positive vessels were analyzed by one-way analysis of variance. The immunopositive rate in extracellular matrix (ECM) staining was analyzed by Pearson’s Chi-square test, and a Tukey–Kramer test was used for post hoc comparisons between the groups. Kaplan–Meier curves were compared using the log-rank statistical method. *p* values of <0.05 was considered to indicate statistical significance.

## 3. Results

### 3.1. Characterization of ADSCs

**Figure 1:**
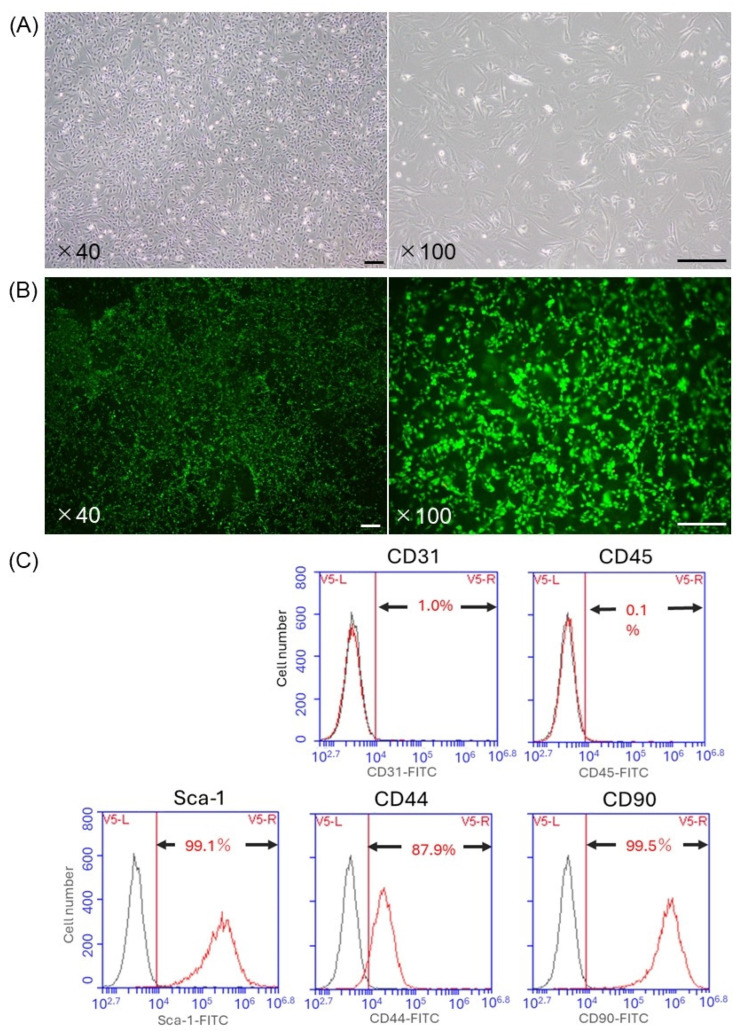
The characteristics of isolated adipose tissue-derived stem cells (ADSCs) originating from C57BL/6 mice. (**A**) The presence of viable ADSCs showing a spindle-shaped morphology. Left: Magnification ×40. Right: Magnification ×100. Calibration bars: 200 µm. (**B**) The viable ADSCs on the RCP device were confirmed by fluorescein diacetate/propidium iodide (FDA/PI) staining. Left: Magnification ×40. Right: Magnification ×100. Calibration bars: 200 µm. (**C**) The expression patterns of cell surface markers of ADSCs were analyzed by flow cytometry. The red lines indicate the results of target-specific antibodies, and the black lines indicate the results of irrelevant antibodies.

ADSCs originating from C57BL/6 mice at passage 1 had a spindle-shaped morphology (Figure 1A). Moreover, FDA/PI staining showed that the ADSCs seeded on the RCP device were viable just before transplantation (Figure 1B). Flow cytometry revealed that the ADSCs at passage 1 were negative for CD31 (1.0%) and CD45 (0.1%) as hematopoietic and endothelial lineage markers, whereas the presence of the stem cell-specific molecules on the cell surfaces was confirmed: Ly6A/E (Sca-1) in 99.1%, CD44 in 87.9%, and CD90 in 99.5% of the cells (Figure 1C).

### 3.2. The Luciferase Expression of Transplanted Bioluminescent ADSCs

**Figure 2:**
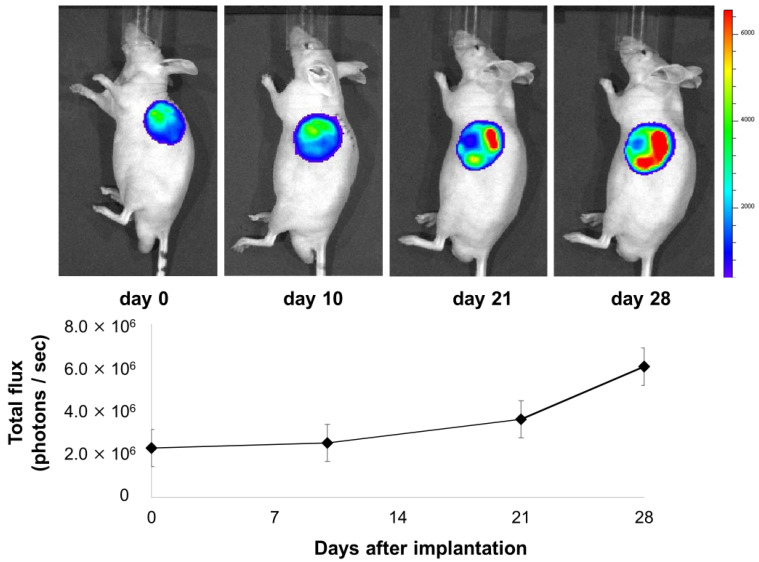
Bioluminescence imaging of transplanted ADSCs originating from Luc-Tg Lewis rats on the RCP device. The presence of viable ADSCs on the RCP device was confirmed until 28 days after implantation into Balb/c nude mice (*n* = 2).

The transplanted ADSCs originating from Luc-Tg Lewis rats on the RCP device were specifically visualized by bioluminescence imaging. The presence of viable ADSCs was confirmed until 28 days after implantation into Balb/c nude mice (*n* = 2). The total flux remained stable until the end of the experiment (Figure 2).

### 3.3. The Outcome of Islet Engraftment after Syngeneic Mouse Islet Transplantation

**Figure 3:**
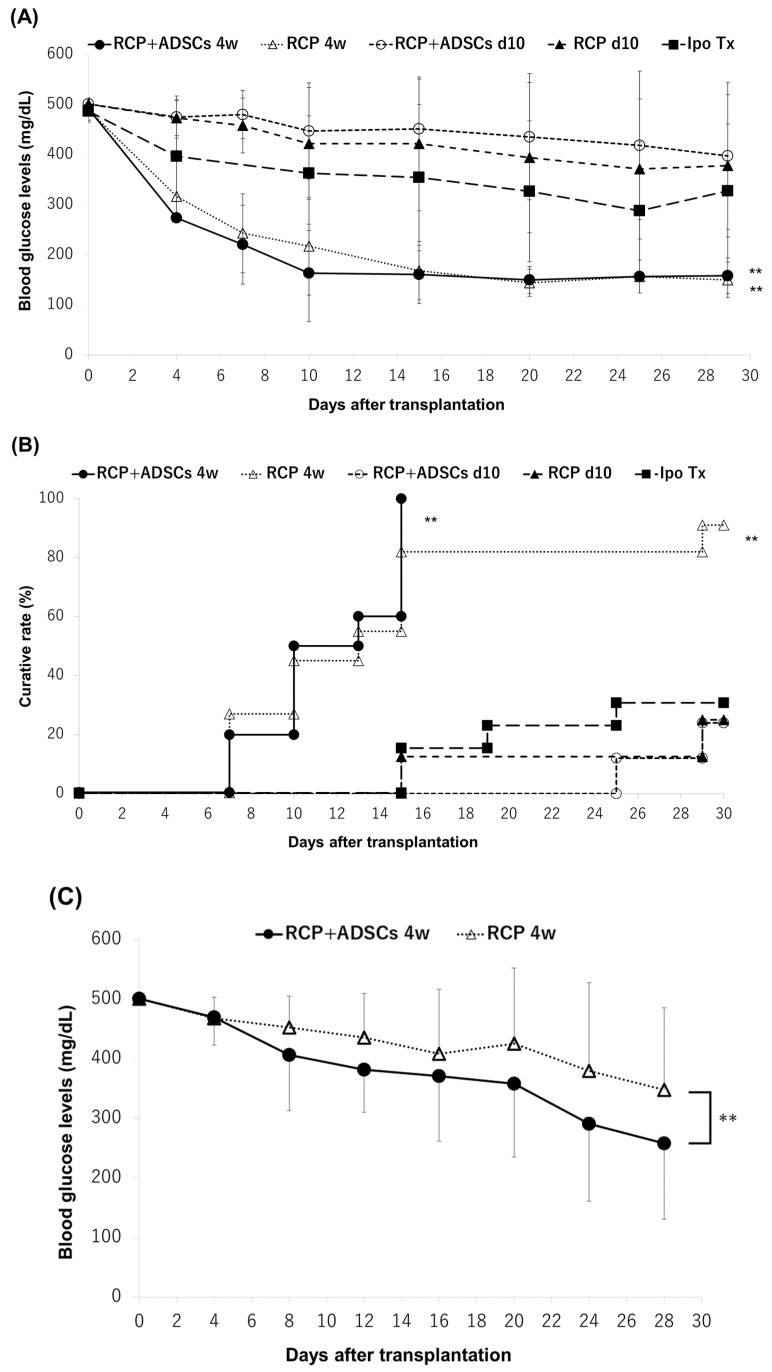

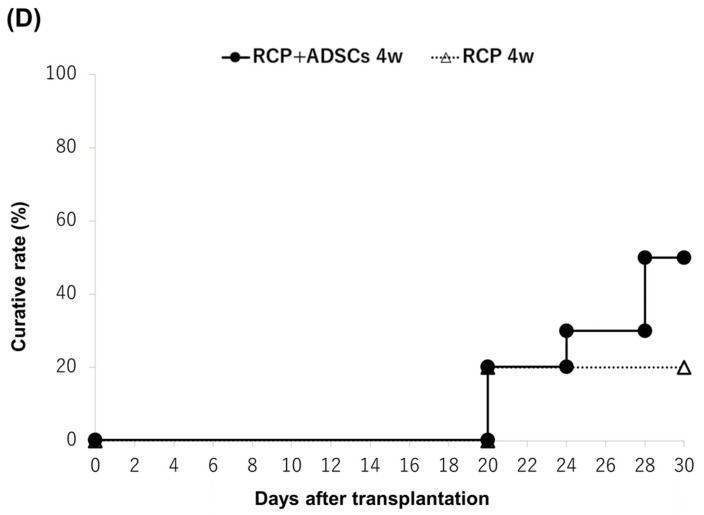
The outcome of islet engraftment after a syngeneic mouse islet transplantation. (**A**) The changes in the blood glucose levels when 300 IEQs of islets were transplanted. (**B**) The number of normoglycemic mice at 30 days when 300 IEQs of islets were transplanted. The RCP+ADSCs-4w (filled circle, *n* = 10) and RCP-4w (open triangle, *n* = 11) groups both showed significantly better blood glucose changes and cure rates (** *p* < 0.01) than the intraportal transplantation (Ipo-Tx) (filled square, *n* = 13) group and RCP+ADSCs-d10 (open circle, *n* = 8) and RCP-d10 (filled triangle, *n* = 8) groups. (**C**) The changes in the blood glucose levels when 120 IEQs of islets were transplanted. The RCP+ADSCs-4w group (open triangle, *n* = 10) showed significantly better blood glucose changes than the RCP-4w group (filled circle, *n* = 10) (** *p* < 0.01). (**D**) The number of normoglycemic mice after 30 days when 120 IEQs of islets were transplanted.

First, we evaluated the efficacy of the engraftment of islets subcutaneously transplanted using an RCP device with/without ADSCs by transplanting a marginal dose of syngeneic mouse islets (300 IEQs). All of the mice in the RCP+ADSCs-4w (*n* = 10) group and 10 of the 11 mice (90.9%) in the RCP-4w group became normoglycemic during the study period. In contrast, the cure rates in both the RCP+ADSCs-d10 and RCP-d10 groups tended to be lower than that in the Ipo-Tx group (25.0% [2/8] and 25.0% [2/8] vs. 30.8% [4/13]). There were no significant differences in the blood glucose changes or cure rates of the RCP+ADSCs and RCP-alone groups (Figure 3A,B). Second, in the RCP+ADSCs-4w and RCP-4w groups, which showed extremely high cure rates when 300 IEQs of islets were transplanted, we evaluated the efficacy of islet engraftment by transplanting a smaller graft volume (120 IEQs). The blood glucose changes in the RCP+ADSCs-4w group (*n* = 10) were significantly ameliorated in comparison to the RCP-4w group (*n* = 10) (*p* < 0.01) (Figure 3C). The cure rate in the RCP+ADSCs-4w group was higher than that in the RCP-4w group (50% [5/10] vs. 20% [2/10]), although the difference did not reach statistical significance (Figure 3D).

### 3.4. Intraperitoneal Glucose Tolerance Test

**Figure 4:**
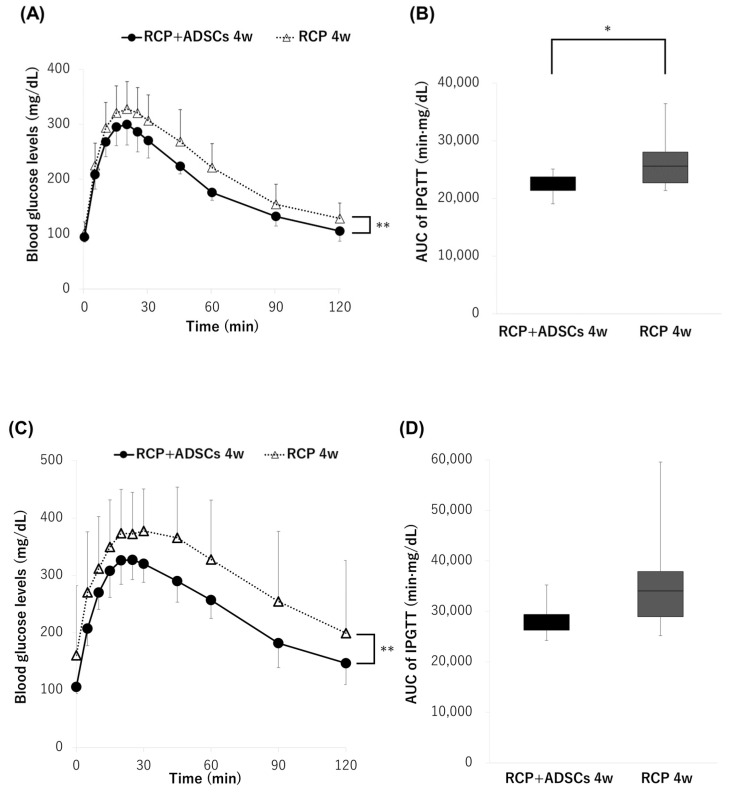
The glucose tolerance profiles of the RCP+ADSCs-4w and RCP-4w groups. (**A**,**B**) The results of the intraperitoneal glucose tolerance test (IPGTT) in the two groups (RCP+ADSCs-4w: filled circle, *n* = 10, RCP-4w: open triangle, *n* = 11) when 300 IEQs of islets were transplanted. Both the blood glucose changes and the area under the curve (AUC) of the IPGTT in the RCP+ADSCs-4w group were significantly ameliorated in comparison to the RCP-4w group (** *p* < 0.01, * *p* < 0.05). (**C**,**D**) The results of the IPGTT in the two groups (RCP+ADSCs-4w: filled circle, *n* = 10, RCP-4w: open triangle, *n* = 10) when 120 IEQs of islets were transplanted. The blood glucose changes of the IPGTT in the RCP+ADSCs-4w group were significantly ameliorated in comparison to the RCP-4w group (** *p* < 0.01).

Although there were no significant differences in the blood glucose changes and cure rates between the RCP+ADSCs-4w and RCP-4w groups when 300 IEQs of islets were transplanted, both the blood glucose changes and the AUC of the IPGTT in the RCP+ADSCs-4w group were significantly ameliorated in comparison to the RCP-4w group (*p* < 0.01, AUC: 22,588 ± 1799 min·mg/dL vs. 26,340 ± 4432 min·mg/dL, *p* < 0.05) (Figure 4A,B). On the other hand, in the RCP+ADSCs-4w and RCP-4w groups, when 120 IEQs of islets were transplanted, the blood glucose changes of the IPGTT in the RCP+ADSCs-4w group were significantly ameliorated in comparison to the RCP-4w group (*p* < 0.01) (Figure 4C). However, there were no significant differences in the AUC of the IPGTT between the RCP+ADSCs-4w and RCP-4w groups (AUC: 36,060 ± 11,661 min·mg/dL vs. 28,443 ± 3730 min·mg/dL) (Figure 4D).

### 3.5. State of Neovascularization and Apoptosis around Transplanted Islet Grafts

**Figure 5:**
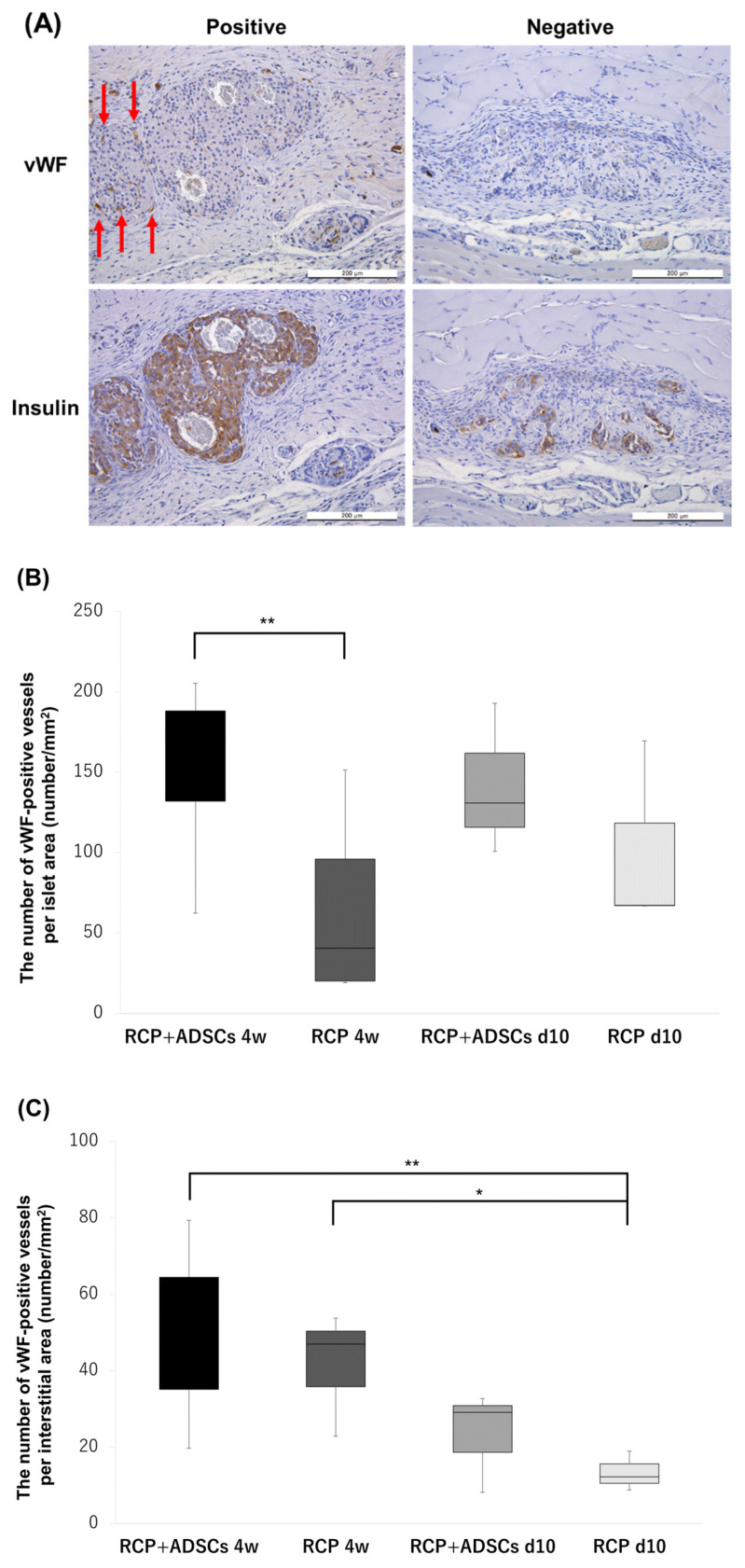
Immunohistochemical analyses to detect neovascularization. (**A**) Upper panel: von Willebrand factor (vWF) staining. Lower panel: insulin staining of the same islets. The red arrows indicate vWF-positive cells. Magnification: ×200. Calibration bars: 200 µm. (**B**) The mean number of new vessels per islet area. The number of vWF-positive vessels in the RCP+ADSCs-4w (*n* = 7) group was significantly higher than that in the RCP-4w (*n* = 6) group (** *p* < 0.01), and that in the RCP+ADSCs-d10 (*n* = 3) group was also higher than that in the RCP-d10 (*n* = 3) group. (**C**) The mean number of new vessels per interstitial area. The number of vWF-positive vessels in the RCP+ADSCs-4w and the RCP-4w groups were significantly higher than that in the RCP-d10 group (** *p* < 0.01, * *p* < 0.05).

Representative examples of insulin and vWF staining are shown in Figure 5A. The number of vWF-positive vessels around islets was counted to examine the state of neovascularization of the islet grafts. In the islet area, the number of vWF-positive vessels in the RCP+ADSCs-4w (*n* = 7) group was significantly higher than that in the RCP-4w (*n* = 6) group (187.8 ± 120.8/mm^2^ vs. 57.1 ± 44.8/mm^2^, *p* < 0.01), and that in the RCP+ADSCs-d10 (*n* = 3) group was also higher than that in the RCP-d10 (*n* = 3) group (141.5 ± 47.0/mm^2^ vs. 101.2 ± 59.2/mm^2^) (Figure 5B). On the other hand, in the interstitial areas, although there were no significant differences in the number of vWF-positive vessels of the RCP+ADSCs-4w and RCP-4w groups, the number of vWF-positive vessels in the RCP+ADSCs-4w and the RCP-4w groups were significantly higher than that in the RCP-d10 group (48.7 ± 20.8/mm^2^, 42.8 ± 12.6/mm^2^ vs. 13.4 ± 5.2/mm^2^, *p* < 0.01, *p* < 0.05) (Figure 5C). TUNEL-positive cells were not observed in any area in any of the groups.

### 3.6. Immunohistochemical staining of Extracellular Matrices

**Figure 6:**
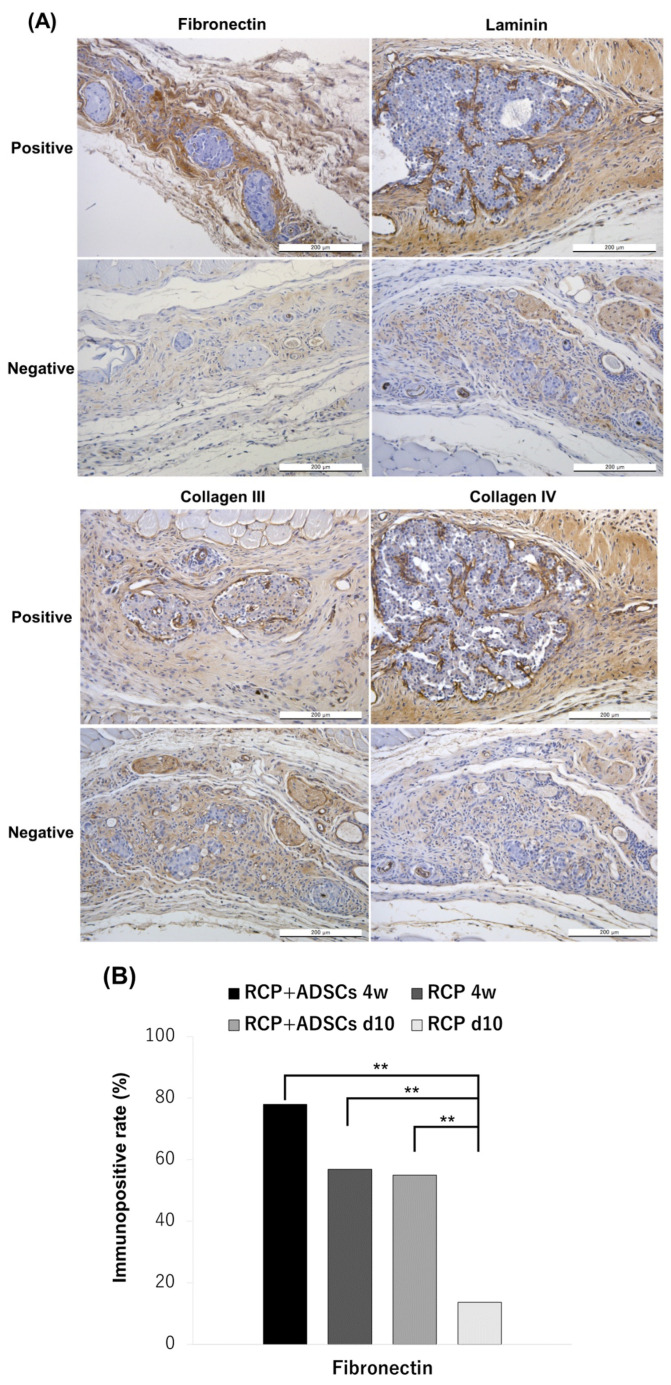

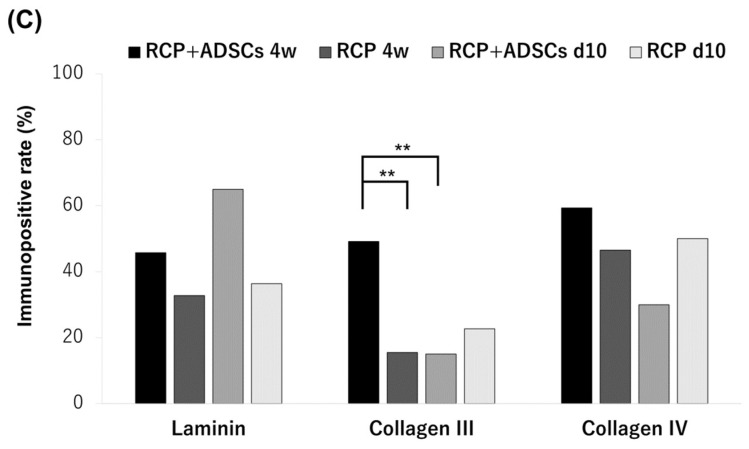
Immunohistochemical staining of extracellular matrices. (**A**) Representative photomicrographs for fibronectin, laminin, collagen III and IV. “Positive” for fibronectin indicates that marked immunopositivity was detectable in the interstitial area. “Positive” for laminin, collagen III and IV indicates that distinct immunopositivity was detectable in the fibrous capsule around the islets. “Negative” indicates that immunopositivity was undetectable. Magnification: ×200. Calibration bars: 200 µm. (**B**) The rate of immunopositive sections in the interstitial area. In fibronectin staining, the rate of immunopositivity in the RCP+ADSCs-4w, RCP-4w and RCP+ADSCs-d10 groups was significantly higher than that in the RCP-d10 group (** *p* < 0.01, ** *p* < 0.01, ** *p* < 0.01, respectively). (**C**) The rate of immunopositive sections in the fibrous capsule around the islets. The rate of collagen III positivity in the RCP+ADSCs-4w group was significantly higher than that of the RCP-4w and ADSCs-d10 groups (** *p* < 0.01, ** *p* < 0.01).

To examine the effect of ECM on subcutaneous islet engraftment, immunohistochemical staining of ECM, including fibronectin, laminin, collagen III, and collagen IV, was performed (Figure 6A). The rate of positive sections in each experimental group was evaluated (Figure 6B,C). In fibronectin staining, the immunopositive rate was significantly higher in the RCP+ADSCs-4w, RCP-4w, and RCP+ADSCs-d10 groups than the RCP-d10 group (*p* < 0.01, *p* < 0.01, *p* < 0.01, respectively). Of particular interest, the rate of collagen III positivity in the islet capsule of the RCP+ADSCs-4w group was also significantly higher than that of the RCP-4w and RCP+ADSCs-d10 groups (*p* < 0.01, *p* < 0.01). The rate of laminin positivity in the RCP+ADSCs groups tended to be higher than the rates in the RCP-alone groups; however, the difference did not reach statistical significance.

## 4. Discussion

Subcutaneous islet transplantation (SC-Tx) has been considered an attractive procedure, because it allows for the easy removal or monitoring of islet grafts with minimal invasiveness and prevents strong innate immune reactions, such as those that occur after intraportal islet transplantation. However, the clinical application of SC-Tx has been challenging due to poor oxygen tension and inadequate vascularization at the subcutaneous transplant site. Thus, the optimization of prevascularization procedures before transplantation is of great importance for improving subcutaneous islet engraftment. In the present study, we demonstrated that the efficacy, in terms of islet engraftment, of SC-Tx using an RCP device is in fact significantly greater than that of standard intraportal islet transplantation in a syngeneic mouse model. Furthermore, we also showed that the addition of ADSCs in combination with an RCP device could enhance subcutaneous islet engraftment, through the restoration of the extracellular matrices (ECMs) in the interstitial parts and the capsule around the islets and the induction of more neovascularization within transplanted islets.

Many studies have reported strategies for inducing neovascularization at subcutaneous transplant sites, including the use of various devices [8,21,22,23], growth factors [24,25] and ECMs [26,27]. Pepper et al. previously demonstrated the successful restoration of euglycemia using a subcutaneous device-less transplant approach in which the tissue under the skin was modified into a highly vascularized engraftment site by temporally exploiting the natural foreign body reaction derived from a nylon catheter [28,29,30]. Although we also had better-than-expected outcomes, in terms of subcutaneous islet engraftment, with a similar procedure, we used an RCP device, which was shown to achieve strong cell adhesion and to efficiently induce neovascularization [12,17]. In addition to the cure rates in the groups in which the RCP devices were placed for four weeks being higher than that in the Ipo-Tx group, it is also noteworthy that almost all of the mice in the four-week RCP device placement became normoglycemic within approximately two weeks when a marginal dose of islets (300 IEQs) was transplanted. Considering that ischemia and hypoxia cannot be prevented for at least 10–14 days until revascularization after intraportal islet transplantation [6], optimized SC-Tx may have great clinical potential. On the other hand, the cure rates in the groups in which the RCP devices were implanted for 10 days before transplantation tended to be lower than that in the Ipo-Tx group. These results indicate that the optimal indwelling period may vary depending on the type of device and animal species. To clarify the optimal indwelling period, further investigations are needed since we did not test the RCP device placement for more than four weeks.

Considering the strong cell adhesion of our RCP device, we applied ADSCs to the RCP device to promote further neovascularization. In comparison to other stem cells, ADSCs can be obtained in large quantities using a relatively simple, non-invasive, and inexpensive procedure. Moreover, the ADSCs secrete various angiogenic growth factors including vascular endothelial growth factor and basic fibroblast growth factor, which can induce the growth and differentiation of endothelial cells. Fumimoto et al. previously reported that they successfully established a rich vascular network in subcutaneous tissue using ADSCs and minced adipose tissue in a mouse model [31]. In the present study, both the blood glucose changes and the glucose tolerance in the RCP+ADSCs-4w group were significantly ameliorated in comparison to the RCP-4w group. Immunohistochemical analyses also showed that the number of vWF-positive vessels within islets in the RCP+ADSCs-4w group was significantly higher than that in the RCP-4w group. In addition, immunohistochemical analyses showed fibronectin and collagen III expression in the RCP+ADSCs-4w group were significantly enhanced in comparison to the RCP-4w group. These findings suggest that the addition of ADSCs in combination with an RCP device could not only induce greater neovascularization within transplanted islets but also enhance the restoration of the ECM and improve the engraftment and function of the islet grafts. These findings are also consistent with previous studies that reported that the intra-islet endothelial cells of transplanted islets can contribute to graft revascularization [32,33].

Immunohistochemistry also showed that the numbers of vWF-positive vessels in both the islet and interstitial areas were higher in the RCP+ADSCs-d10 group than in the RCP-d10 group, though the functionality of identified vessels remains uncertain. However, there were no significant differences in islet engraftment or function between the two groups. In addition, although the RCP+ADSCs-4w group showed excellent islet engraftment and function compared with the RCP+ADSCs-d10 group, the number of vWF-positive vessels was almost the same between the two groups. These contradictions may suggest, as previously described [12], that not only neovascularization but also the enhancement and/or compensation of the ECM is an important factor for improving subcutaneous islet engraftment. In other words, the enhancement and/or compensation of the ECM led by temporary inflammation derived from foreign body reactions might have been insufficient in the groups with the 10-day placement of RCP devices in comparison to the groups with 4-week placement. To confirm this hypothesis, we performed immunohistochemical staining of ECM components, including laminin, fibronectin, collagen III, and collagen IV. First, we evaluated the interstitial areas around the islets by fibronectin staining. The RCP-4w group showed a significantly higher rate of immunopositivity than the RCP-d10 group. This finding suggests that the four-week placement of the RCP device could promote scaffold construction as a neovascularization bed for improving subcutaneous islet engraftment. This hypothesis is consistent with a previous study that reported that slight inflammation had the potential to increase the expression of fibronectin and extend the survival time of stem cells [34]. Secondly, we evaluated the fibrous capsular around islets by laminin, collagen III, and collagen IV staining. Notably, the rate of collagen III immunopositivity in the RCP+ADSCs-4w group was significantly higher than that in the RCP-4w and RCP+ADSCs-d10 groups. This result indicates that the four-week placement of the RCP device could enhance the restoration of the fibrous capsule surrounding islet grafts only when combined with ADSCs. In particular, several studies have reported that among the ECM components, collagen III is crucial for maintaining the function of islet grafts [35,36]. Moreover, the rate of laminin, collagen III, and collagen IV immunopositivity in the RCP+ADSCs-4w group was higher than that in the RCP-4w group. This finding suggests that the addition of ADSCs in combination with an RCP device could improve the islet function by enhancing these ECM components surrounding islet grafts.

Taken together, to improve the engraftment and function of islet grafts which have been degraded by the islet isolation procedure, it is significant to induce more neovascularization and enhance the restoration of the ECM in the islet grafts and the interstitial parts surrounding islet grafts. We demonstrated that the four-week placement of an RCP device mainly contributed to enhancing ECM restoration as scaffold construction, and the addition of ADSCs in combination with an RCP device contributed to both inducing neovascularization and enhancing ECM restoration in the islet grafts. However, to clarify the mechanisms through which the addition of ADSCs benefits islet engraftment, further investigations are needed since a sham group was removed from the present study due to the obvious inferior outcome in our previous study [12]. Furthermore, we would like to test the RCP device combined with ADSCs in different animal strains or species for clinical application.

## 5. Conclusions

We report that a novel prevascularization procedure using an RCP device combined with ADSCs has great potential to improve the engraftment and function of islets in comparison to the current standard method of intraportal islet transplantation. The further optimization of the present procedure may enable the successful clinical application of a single-donor-to-single-recipient model of islet transplantation.

## Data Availability

All data generated or analyzed in the present study are included in this manuscript.
